# Complete genome sequence of *Kosakonia oryzae* type strain Ola 51^T^

**DOI:** 10.1186/s40793-017-0240-8

**Published:** 2017-04-17

**Authors:** Yuanyuan Li, Shuying Li, Mingyue Chen, Guixiang Peng, Zhiyuan Tan, Qianli An

**Affiliations:** 1 State Key Laboratory of Rice Biology, Institute of Biotechnology, Zhejiang University, Hangzhou, China; 2 College of Natural Resources and Environment, South China Agricultural University, Guangzhou, 510642 China; 3 College of Agriculture, South China Agricultural University, Guangzhou, 510642 China

**Keywords:** Endophyte, *Kosakonia*, Nitrogen fixation, Plant growth-promoting bacteria

## Abstract

Strain Ola 51^T^ (=LMG 24251^T^ = CGMCC 1.7012^T^) is the type strain of the species *Kosakonia oryzae* and was isolated from surface-sterilized roots of the wild rice species *Oryza latifolia* grown in Guangdong, China. Here we summarize the features of the strain Ola 51^T^ and describe its complete genome sequence. The genome contains one circular chromosome of 5,303,342 nucleotides with 54.01% GC content, 4773 protein-coding genes, 16 rRNA genes, 76 tRNA genes, 13 ncRNA genes, 48 pseudo genes, and 1 CRISPR array.

**Electronic supplementary material:**

The online version of this article (doi:10.1186/s40793-017-0240-8) contains supplementary material, which is available to authorized users.

## Introduction

*Enterobacter cowanii* [1], *E. radicincitans* [2], *E. oryzae* [3], *E. arachidis* [4], *E. sacchari* [5], *E. oryziphilus* [6, 7], and *E. oryzendophyticus* [6, 7] have been transferred into the novel genus *Kosakonia* of the family “*Enterobacteriaceae*” [8–10]. A novel species “*Kosakonia pseudosacchari”* [11] closely related to *K. sacchari* was recently proposed. With the exception of the type species *K. cowanii* *,* which was originally obtained from clinical samples [1], the other members of the genus *Kosakonia* are nitrogen-fixing bacteria associated with plants [2–6, 11] and commonly occur in the nitrogen-fixing bacterial community of some non-legume crops, such as rice [6] and sugarcane [12]. Some nitrogen-fixing *Kosakonia* strains are able to promote crop growth [12–14].

Strain Ola 51^T^ (=LMG 24251 ^T^=CGMCC 1.7012 ^T^) is the type strain of the species *Kosakonia oryzae* and was isolated from surface-sterilized roots of the wild rice species *Oryza latifolia* grown in Guangdong, China [3]. Here we present the summary of the features of the *K. oryzae* type strain Ola 51^T^ and its complete genome sequence, which provides a reference for resolving the phylogeny and taxonomy of closely related strains and the genetic information to study its plant growth-promoting potential and its plant-associated life style.

## Organism information

### Classification and features

**Fig. 1:**
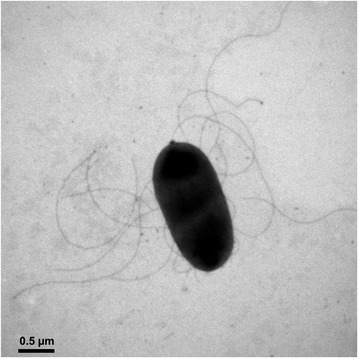
Cell morphology of the *Kosakonia oryzae* type strain Ola 51^T^. The bacterium was stained by uranyl acetate and observed by a transmission electron microscope

*K. oryzae* strain Ola 51^T^ is a Gram-negative, non-spore-forming, motile rod with peritrichous flagella (Fig. 1). It grows aerobically but reduces N_2_ to NH_3_ at a low pO_2_. It forms circular, convex, smooth colonies with entire margins on nutrient agar [3, 8]. It grows best around 30 °C and pH 7 (Table 1) [3]. *K. oryzae* Ola 51^T^ has the typical biochemical phenotypes of the genus *Kosakonia*: positive for acetoin production (Voges-Proskauer test) while negative for indole production; positive for β-galactosidase and arginine dihydrolase while negative for lysine decarboxylase; positive for oxidation of arabinose, cellobiose, citrate, fructose, galactose, gluconate, glucose, glycerol, lactose, malate, maltose, mannitol, mannose, sorbitol, sucrose and trehalose (Table 1) [3, 8].

**Table 1:** Classification and general features of *Kosakonia oryzae* strain Ola 51^T^ according to the MIGS recommendations [15]

MIGS ID	Property	Term	Evidence code^a^
	Classification	Domain *Bacteria*	TAS [34]
		Phylum *Proteobacteria*	TAS [35]
		Class *Gammaproteobacteria*	TAS [36, 37]
		Order “*Enterobacteriales*”	TAS [38]
		Family *Enterobacteriaceae*	TAS [39, 40]
		Genus *Kosakonia*	TAS [8]
		Species *Kosakonia oryzae*	TAS [3, 8]
		Type strain: Ola 51^T^	TAS [3]
	Gram stain	Negative	TAS [3]
	Cell shape	Rod	TAS [3]
	Motility	Motile	TAS [3]
	Sporulation	Non-sporulating	TAS [3]
	Temperature range	10–40 °C	TAS [3]
	Optimum temperature	28–37 °C	TAS [3]
	pH range; Optimum Carbon source	3.5–10; 6.0–8.0 Arabinose, cellobiose, citrate, fructose, galactose, gluconate, glucose, glycerol, lactose, malate, maltose, mannitol, mannose, sorbitol, sucrose & trehalose	TAS [3]
			TAS [3, 8]
MIGS-6	Habitat	Plants	TAS [3]
MIGS-6.3	Salinity	0 – 5% NaCl (w/v)	TAS [3]
MIGS-22	Oxygen requirement	Facultatively anaerobic	TAS [3]
MIGS-15	Biotic relationship	Free-living, endophytic	TAS [3]
MIGS-14	Pathogenicity	Not reported	
MIGS-4	Geographic location	Guangzhou, Guangdong, China	TAS [3]
MIGS-5	Sample collection	September 12, 2005	TAS [3]
MIGS-4.1 MIGS-4.2	Latitude	23.1634171311 °N	NAS
	Longitude	113.3534469581°E	NAS
MIGS-4.3	Depth	0.2 – 0.3 m below the surface	TAS [3]
MIGS-4.4	Altitude	20 m	NAS

^a^ Evidence codes – IDA: Inferred from Direct Assay; TAS: Traceable Author Statement (i.e., a direct report exists in the literature); NAS: Non-traceable Author Statement (i.e., not directly observed for the living, isolated sample, but based on a generally accepted property for the species, or anecdotal evidence). These evidence codes are from the Gene Ontology project [41]

**Fig. 2:**
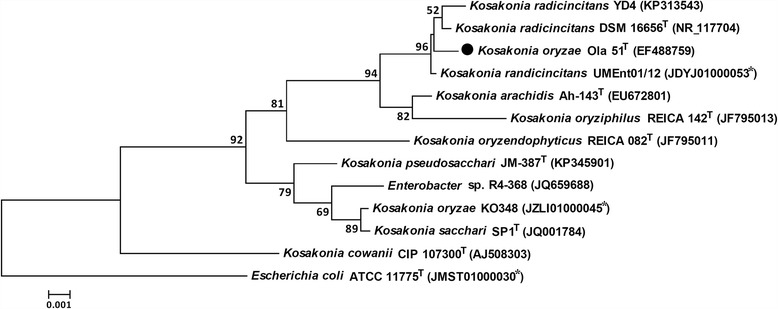
Phylogenetic tree based on the 16S rRNA gene sequences showing the phylogenetic position of the *Kosakonia oryzae* type strain Ola 51^T^ (●) and other strains belonging to the genus *Kosakonia*. The sequences were aligned using the SINA (SILVA Incremental Aligner) Alignment Service [42] and were constructed to the phylogenetic tree with the neighbor-joining algorithm and the Kimura 2-parameter model integrated in the MEGA 5.2 program [43]. Bootstrap values (>50%) of 1,000 tests are shown at the nodes. The tree was rooted on the outgroup *Escherichia coli* ATCC 11775^T^. The GenBank accession numbers of the sequences are indicated in brackets; * indicates the accession number of a contig of the whole genome sequence. The scale bar indicates 0.1% substitutions per site

The 16S rRNA gene sequence of *K. oryzae* Ola 51^T^ was deposited in GenBank under the accession number EF488759 [3]. A phylogenetic analysis of the 16S rRNA gene sequences from the strains belonging to the genus *Kosakonia* and *Escherichia coli* ATCC11775 ^T^ (the type strain of the type species of the type genus of the family *Enterobacteriaceae*) showed that *K. oryzae* Ola 51^T^ is most closely related to the strains belonging to the species *K. radicincitans* (Fig. 2) [3, 8–11].

### Chemotaxonomic data

Whole-cell fatty acids were extracted from cells grown aerobically at 28 °C for 24 h on the TSA medium according to the recommendations of the Microbial Identification System (MIDI Inc., Delaware USA). The whole-cell fatty acid composition was determined using a 6890 N gas chromatograph (Agilent Technologies, Santa Clara, USA) and the peaks of the profiles were identified using the TSBA50 identification library version 5.0 (MIDI). *K. oryzae* Ola 51^T^ shows the typical cell fatty acid profile of the genus *Kosakonia* [8]. The major fatty acids are C_16:0_, C_18:1 ω7c_, C_16:1 ω7c/15:0 iso 2OH_, C_17:0 cyclo_ and C_14:0 3OH/16:1 iso I_ [8, 11].

## Genome sequencing information

### Genome project history

*K. oryzae* Ola 51^T^ was selected for sequencing based on its taxonomic significance. The genome sequence is deposited in GenBank under the accession number CP014007. A summary of the genome sequencing project information and its association with MIGS version 2.0 [15] is shown in Table 2.

**Table 2:** Genome sequencing project information for *Kosakonia oryzae* strain Ola 51^T^

MIGS ID	Property	Term
MIGS 31	Finishing quality	Finished
MIGS-28	Libraries used	PacBio 8 –11 Kb library
MIGS 29	Sequencing platforms	PacBio RS II
MIGS 31.2	Fold coverage	PacBio 128 ×
MIGS 30	Assemblers	HGAP Assembly.3 in SMRT analysis-2.3.0
MIGS 32	Gene calling method	GeneMarkS+
	Locus Tag	AWR26
	Genbank ID	CP014007
	GenBank Date of Release	June 6, 2016
	GOLD ID	Gp0154734
	BIOPROJECT	PRJNA309028
MIGS 13	Source Material Identifier	LMG 24251^T^ = CGMCC 1.7012^T^
	Project relevance	Taxonomy, agriculture, plant-microbe interactions

### Growth conditions and genomic DNA preparation

*K. oryzae* Ola 51^T^ was grown aerobically in liquid Luria-Bertani medium at 30 °C until early stationary phase. The genome DNA was extracted from the cells by using a TIANamp bacterial DNA kit (Tiangen Biotech, Beijing, China). DNA quality (OD260/OD280 = 1.8) and quantity (22 μg) were determined with a Nanodrop spectrometer (Thermo Scientific, Wilmington, USA).

### Genome sequencing and assembly

The genomic DNA of *K. oryzae* Ola 51^T^ was constructed into 8 – 11 kb insert libraries and sequenced using PacBio SMRT sequencing technology [16] at the Duke University Genome Sequencing & Analysis Core Resource. Sequencing was run on two SMRT cells and resulted in 124,997 high-quality filtered reads with an average length of 8,260 bp. High-quality reads were assembled by the RS_HGAP_Assembly.3 in the SMRT analysis v2.3.0. The final assembly produced 128-fold coverage of the genome.

### Genome annotation

Automated genome annotation was done using the NCBI Prokaryotic Genome Annotation Pipeline [17]. Functional annotations were done by searching against the KEGG [18], InterPro [19], and COG [20] databases. Genes with signal peptides were predicted using SignalP [21]. Genes with transmembrane helices were predicted using TMHMM [22].

## Genome properties

**Fig. 3:**
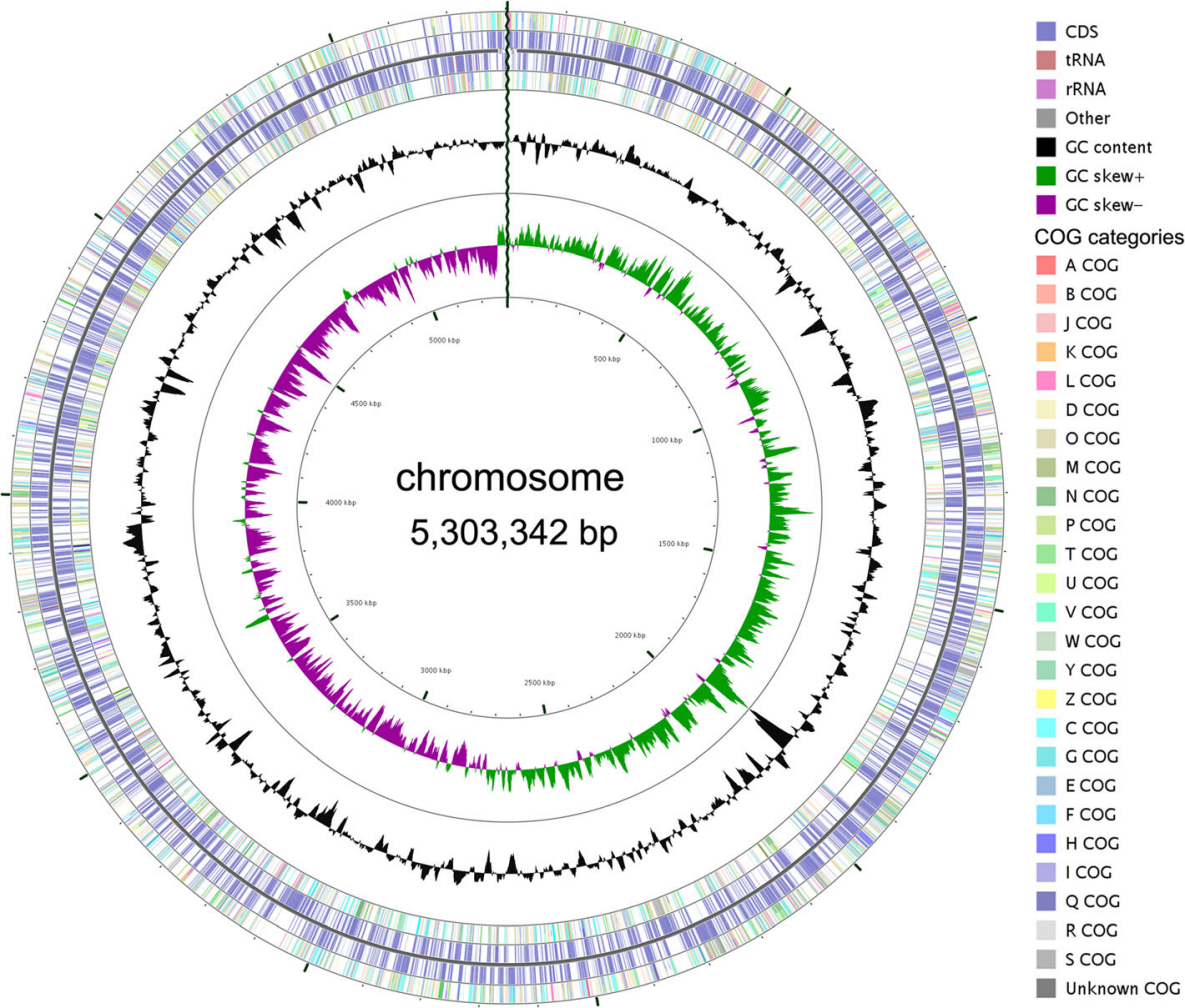
Circular map of the chromosome of the *Kosakonia oryzae* strain Ola 51^T^. From outside to the center: CDS on forward strand colored according to their COG categories (oranges/reds: information storage and processing; greens/yellows: cellular processes and signaling; blues/purples: metabolism; grays: pooly characterized), CDS and RNA genes on forward strand, CDS and RNA genes on reverse strand, CDS on reverse strand colored according to their COG categories, GC content, and GC skew. The circular map was generated by CGView [44]

The genome of *K. oryzae* Ola 51^T^ contains one circular chromosome (Fig. 3). The chromosome contains 5,303,342 nucleotides with 54.0% G + C content. The genome contains 4,926 predicted genes, 4773 protein-coding genes, 105 RNA genes (16 rRNA genes, 76 tRNA genes, and 13 ncRNA genes), 48 pseudo genes, and 1 CRISPR repeats. Among the 4,773 protein-coding genes, 3,765 genes (78.88%) have been assigned functions, while 1008 genes (21.12%) have been annotated as hypothetical or unknown proteins (Table 3). The distribution of genes into COG functional categories is presented in Table 4 and Fig. 3.

**Table 3:** Genome statistics

Attribute	Value	% of Total
Genome size (bp)	5,303,342	100
DNA coding (bp)	4,613,400	86.99
DNA G + C (bp)	2,864,594	54.01
DNA scaffolds	1	100
Total genes	4,926	100
Protein-coding genes	4,773	96.89
RNA genes	105	2.13
Pseudo genes	48	0.97
Genes in internal clusters	ND	
Genes with function prediction	3765	76.43
Genes assigned to COGs	4237	86.01
Genes with Pfam domains	4416	89.65
Genes with signal peptides	432	8.77
Genes with transmembrane helices	1179	23.93
CRISPR repeats	1	0.02

**Table 4:** Number of genes associated with general COG functional categories

Code	Value	%age	Description
J	194	4.06	Translation, ribosomal structure and biogenesis
A	1	0.02	RNA processing and modification
K	414	8.67	Transcription
L	140	2.93	Replication, recombination and repair
B	0	0	Chromatin structure and dynamics
D	35	0.73	Cell cycle control, Cell division, chromosome partitioning
V	60	1.26	Defense mechanisms
T	278	5.82	Signal transduction mechanisms
M	270	5.66	Cell wall/membrane biogenesis
N	163	3.42	Cell motility
U	123	2.58	Intracellular trafficking and secretion
O	154	3.23	Posttranslational modification, protein turnover, chaperones
C	287	6.01	Energy production and conversion
G	428	8.97	Carbohydrate transport and metabolism
E	476	9.97	Amino acid transport and metabolism
F	93	1.95	Nucleotide transport and metabolism
H	188	3.94	Coenzyme transport and metabolism
I	152	3.18	Lipid transport and metabolism
P	293	6.14	Inorganic ion transport and metabolism
Q	98	2.05	Secondary metabolites biosynthesis, transport and catabolism
R	502	10.52	General function prediction only
S	422	8.84	Function unknown
-	536	11.23	Not in COGs

The total is based on the total number of protein coding genes in the genome

## Insights from the genome sequence

The genome sequences of *K. cowanii* JCM 10956 ^T^, *K. radicincitans* DSM 16656 ^T^ (=D5/23^T^) [23], *K. radicincitans* UMEnt01/12 [24], *K. radicincitans* YD4 [25], *K. sacchari* SP1^T^ [26], *“K. pseudosacchari”* JM-387^T^ [11], *K. oryzae* KO348 [27], and *Enterobacter* sp. R4-368 [28] which was close to *K. sacchari* SP1^T^ [26] had been deposited in the GenBank database. The genome ANIs (Additional file 1: Table S1) between Ola 51^T^ and the other strains belonging to the genus *Kosakonia* were calculated using the Orthologous Average Nucleotide Identity tool [29]. The cut-off ANI value for species boundary was set at 95% - 96% [30]. The ANI value (95.85%) between *K. oryzae* Ola 51^T^ and *K. radicincitans* DSM 16656 ^T^ is in the fuzzy zone 95% - 96%. The digital DDH value between Ola 51^T^ and DSM 16656 ^T^ calculated by the Genome-to-Genome Distance Calculator [31] with the Formula 2 is 66.2%, below the 70% cut-off value for species boundary. Moreover, Ola 51^T^ and DSM 16656 ^T^ were differentiated by metabolic phenotypes [3, 11] and ribosomal protein mass profiles [5]. Therefore, *K. oryzae* and *K. radicincitans* are closely related sister species.

Strain YD4 was closer to *K. radicincitans* DSM 16656 ^T^ than *K. oryzae* Ola 51^T^ on the phylogenetic tree based on the 16S rRNA genes (Fig. 2). However, the ANI value and the digital DDH value between YD4 and *K. radicincitans* DSM 16656 ^T^ is 95.56% and 64.4%, respectively, while between YD4 and *K. oryzae* Ola 51^T^ is 97.04% and 74.3%, respectively. Therefore, the strain YD4 belongs to *K. oryzae* but not *K. radicincitans*.

Strain KO348 was grouped with *K. sacchari* SP1^T^, *Enterobacter* sp. R4-368, and *“K. pseudosacchari”* JM-387^T^ on the phylogenetic tree based on the 16S rRNA genes (Fig. 2). The ANI value between KO348 and *K. oryzae* Ola 51^T^ is 84.04%. The strain KO348 thus does not belong to *K. oryzae*. The ANI value between KO348 and *Enterobacter* sp. R4-368 [27], *K. sacchari* SP1^T^, or *“K. pseudosacchari”* JM-387^T^ is 98.80%, 94.56%, or 94.05%, respectively. Therefore, KO348 and R4-368 belong to the same species, likely a novel species closely related to *K. sacchari* and *“K. pseudosacchari”*.

*K. oryzae* Ola 51^T^ and YD4, *K. radicincitans* DSM 16656 ^T^ and UMEnt01/12, *K. sacchari* SP1^T^, *“K. pseudosacchari”* JM-387^T^, and *Kosakonia* sp. KO348 and R4-368 were all isolated from plants. Their genomes contain genes encoding multiple enzymes degrading plant cell wall polysaccharides and removing reactive oxygen species, likely facilitating endophytic colonization [32]. They all contain genes encoding the regulatory protein (Fha1) and structural proteins (Lip, IcmF, DotU and ClpV) and secreted proteins (VgrG and Hcp) of the type VI secretion system, which may play a role in the plant-associated lifestyle [32]. Except *K. radicincitans* DSM 16656 ^T^ and UMEnt01/12, these strains contain the most structural proteins (YscCJRSTUVN) of the type III secretion system, which is not widespread among the previously studied endophytic bacteria [32].

These plant-associated *Kosakonia* strains contain genes contributing to multiple plant growth-promoting activities. They all contain the *nif* gene cluster (*nifJHDKTYENXUSVWZMFLABQ*) for the Mo-Fe nitrogenase-dependent nitrogen fixation, the genes encoding indole-3-acetaldehyde dehydrogenase, aspartate aminotransferase, aromatic amino acid aminotransferase and phenylpyruvate decarboxylase for producing the phytohormone auxin, and the *budABC* genes for producing volatile acetoin and 2,3-butanediol which induce plant systemic resistance to pathogens [33]. In addition, *K. oryzae* Ola 51^T^ and YD4, and *K. radicincitans* DSM 16656 ^T^ and UMEnt01/12 also contain the *anf* gene cluster (*anfHDGK*) for the Fe-Fe nitrogenase-dependent nitrogen fixation. In contrast, the clinical strain *K. cowanii* JCM 10956 ^T^ does not contain the *nif* gene cluster.

## Conclusions

The phylogeny of the members of the genus *Kosakonia* based on the 16S rRNA gene sequences is roughly in agreement with their overall genome relatedness. The complete genome sequence of *K. oryzae* Ola 51^T^ provides the reference genome for genomic identification of strains belonging to *K. oryzae*. Analyses of the overall genome relatedness indices (ANI and digital DDH values), easily and reliably show that *K. oryzae* and *K. radicincitans* are closely related sister species and that the strain YD4, which shows close 16S rRNA gene-based phylogeney to *K. radicincitans* and was classified into *K. radicincitans*, belongs to *K. oryzae*. As well as YD4, which is able to promote growth of the yerba mate plants in low-fertility soils [14], *K. oryzae* Ola 51^T^ contains both the *nif* gene cluster and the *anf* gene cluster for nitrogen fixation and genes contributing to production of auxin and volatile acetoin and 2,3-butanediol. Therefore, *K. oryzae* Ola 51^T^ may be able to promote plant growth. Genomic analyses also show that *K. oryzae* Ola 51^T^ and YD4 may have the type III and VI secretion systems and thus motivate us to study the functions of the type III and VI secretion systems in the interactions between beneficial *Kosakonia* bacteria and plants.

## Additional file

Additional file 1: Table S1. Average nucleotide identities (ANIs) between genomes of the strains belonging to the genus *Kosakonia* *.* (DOC 38 kb)

## Abbreviations

ANI: Average nucleotide identity
DDH: DNA-DNA hybridization
SMRT: Single Molecule Real-Time
