# Supplementary material for: Complete genome sequence of Kosakonia oryzae type strain Ola 51T
Source: Stand Genomic Sci. 2017 Apr 17;12:28. doi: 10.1186/s40793-017-0240-8 (PMC5392936; doi:10.1186/s40793-017-0240-8)
Supplement: Additional file 1: Table S1. — Average nucleotide identities (ANIs) between genomes of the strains belonging to the genus Kosakonia . (DOC 38 kb) [file 40793_2017_240_MOESM1_ESM.doc]

***Table S1****. Average nucleotide identities (ANI) between genomes of the strains belonging to the genus Kosakonia*

| **Strain**  (accession number) | **ANI values (%)** | | | | | | | | |
| --- | --- | --- | --- | --- | --- | --- | --- | --- | --- |
|  | Ola51^T^ | YD4 | DSM 16656^T^ | UMEnt01/12 | KO348 | R4-368 | SP1^T^ | JM-387^T^ | JCM 10956^T^ |
| *K. oryzae* Ola51^T^  (CP014007.1) |  | **97.04**^a^ | 95.92 | 95.85 | 84.04 | 84.10 | 83.96 | 83.82 | 82.39 |
| *K. randicincitans* YD4  (JSFC00000000.1) |  |  | 95.56 | 95.54 | 84.26 | 84.28 | 84.06 | 83.94 | 82.52 |
| *K. randicincitans* DSM 16656^T^  (AKYD00000000.1) |  |  |  | **99.12**^a^ | 84.03 | 83.97 | 83.83 | 83.83 | 82.28 |
| *K. randicincitans* UMEnt01/12  (JDYJ00000000.1) |  |  |  |  | 84.13 | 84.02 | 83.89 | 83.83 | 82.31 |
| *K. oryzae* KO348  (JZLI00000000.1) |  |  |  |  |  | **98.80**^a^ | 94.56 | 94.05 | 82.89 |
| *Enterobacter* sp. R4-368  (CP005991.1; CP005992.1) |  |  |  |  |  |  | 94.44 | 93.90 | 82.84 |
| *K. sacchari* SP1^T^  (CP007215.3) |  |  |  |  |  |  |  | 94.75 | 82.66 |
| *K. pseudosacchari* JM-387^T^  (ERR996272) |  |  |  |  |  |  |  |  | 82.77 |
| *K. cowanii* JCM 10956^T^  (BBEU00000000.1) |  |  |  |  |  |  |  |  |  |

^a^ANI values >96% are indicated in bold.
